# Perceived performances of peer learning and education approach on malaria prevention through primary schools communities in rural Ethiopia: Peer educators' perspectives

**DOI:** 10.3389/fpubh.2022.861253

**Published:** 2022-11-15

**Authors:** Alemayehu Deressa, Addis Eyeberu, Mulugeta Gamachu, Adera Debella, Fira Abamecha Ababulgu

**Affiliations:** ^1^Department of Public Health and Policy, School of Public Health, Haramaya University, Harar, Ethiopia; ^2^School of Nursing and Midwifery, College of Health and Medical Sciences, Haramaya University, Harar, Ethiopia; ^3^Department of Health, Behavior, and Society, Faculty of Public Health Institute of Health, Jimma University, Jimma, Ethiopia

**Keywords:** peer learning, peer educators, malaria prevention, school, Ethiopia

## Abstract

**Background:**

Schools are viewed as natural hubs and an effective strategy for promoting community healthy practices such as malaria prevention and control. This study examined the perceived performance of the peer learning and education approach to malaria prevention in rural primary school communities in Ethiopia, which has thus far received little attention.

**Methods:**

Post-intervention data were collected from 404 randomly selected peer educators between 2 April and June 2020 using a structured questionnaire. The data were analyzed using the Statistical Package for Social Sciences (SPSS) version 24.0. Multiple linear regression was used to identify independent predictors of perceived performance of school-based peer learning and educational approach. A statistically significant *p*-value of < 0.05 was considered statistically significant.

**Results:**

Four hundred and one educators (99.2%) completed the interview. The mean score of perceived performance was 44.31 (SD = 6.13) which was above the expected mean level range of 11–55. Feasibility (β = 0.253, 95% CI = [0.313, 0.682]), and appropriateness (β = 0.163, 95% CI = [0.099, 0.442]) were significantly associated with perceived performance. Self-efficacy, malaria risk perception, eagerness to share and learn from each other's experiences, and preference for more than one subject were all significantly associated with perceived performance, with (β = 0.097, CI = [0.017, 0.242]), (β = 0.143, CI = [0.071, 0.233]), (β = 0.207, CI = [0.308, 0.826]) and (β = 0.075, CI = [0.084, 2.511]) at 95% CI, respectively.

**Conclusions:**

The peer learning and education approach to malaria prevention and control in schools has a noticeably high level of perceived performance. Thus, it is recommended that when designing and implementing such programs through schools, personal and social (team) factors such as self-efficacy, risk perceptions, and peer education team spirit be considered.

## Background

Malaria is the most important public health issue causing health, economic and social crises worldwide (1). Between 2016 and 2017, Africa bore the heaviest burden of malaria, with an estimated 451,000 deaths from malaria (2, 3). In Ethiopia, there were 1,530,739 confirmed malaria cases, with 356 reported deaths (3). According to the World Health Organization's most recent World Malaria Report, 241 million malaria cases and 627,000 malarial deaths were estimated worldwide in 2020. This translates to an additional 14 million in 2020 compared to 2019, as well as 69,000 more deaths. During the COVID-19 pandemic, approximately two-thirds of these additional deaths (47,000) were attributed to disruptions in malaria prevention, diagnosis, and treatment (4).

Malaria affects almost all people who live in malaria-prone areas. Children and pregnant women are the most vulnerable groups (5). Malaria affects directly through infection and indirectly it causes co-morbidity such as anemia and other nutritional deficiencies (3). Children with malarial infection are more likely to be stunted and wasted, which results in 16% of all repetitions in primary school children being associated with stunting (6). A recent study showed that malaria was the first cause of school absenteeism in Africa (7). Despite these alarming realities, evidence shows that the adoption of preventive methods is poor. The use of preventive methods is determined by different factors such as the perception of causes and disease transmission, mosquito prevalence, affordability, and climate (4, 5).

Furthermore, malaria continues to be a major public health issue among school-aged children, affecting a critical period of learning and development (8, 9). Less emphasis is given to school-aged children for malaria control, yet the prevalence of Plasmodium infection in this age group often exceeds infections seen in much younger children (8, 10). For instance, the overall prevalence of Plasmodium infection in Oromia, Ethiopia was found to be 0.56% (with 53% of infections due to Falciparum and 47% due to P. vivax). The prevalence of anemia among the children surveyed was 17.6% in 2011 (11).

To this effect, the Federal Ministry of Health (FMOH) emphasized the need for malaria prevention and control, and targeted global malaria elimination by 2030 (12, 13). Evidence indicates that there are gaps in knowledge, attitude, health-seeking behaviors, and the use of preventive measures among school students with regard to malaria (14, 15). These gaps can be reduced when adequate emphasis is given to peer learning and education on malaria prevention and control in schools (16).

School health initiatives have encompassed strategies aimed at improving the capacity, knowledge, and decision-making skills that help to promote health and prevent diseases among school children and their families (17–19). In this regard, school students were also perceived to play a pivotal role in keeping the health of their families and communities. Malaria is one of the focal topics of school health programs globally (20–23). School Health Malaria Control has been associated with a significant reduction in malaria-related morbidity and mortality as well as improvements in educational outcomes including improved school examination scores (16).

Indeed, peer health education has been in use in developed and developing countries and studies have reported its efficacy in changing the beliefs of students that enhance their performance in the disease prevention and control (24). Few studies have reported peer educators gaining more knowledge and skills that decreased their high-risk behavior (25), benefited experience as educators that lead to attitude and behavior change (26), and overall gains in the three domains of cognitive and behavioral, connectedness and self-concept, and information changes.

Despite its wide use, perceptions of peer educators on peer education have not been reported (15, 27, 28). So, it can be applied as a complementary approach to existing malaria control strategies activities. Insights on how to effectively engage primary school students in promoting malaria preventive actions both in schools and the communities remain unexplored. This study evaluated the perceived outcomes of peer learning and education approach on malaria prevention (PLEA-Malaria) in rural primary school communities in Ethiopia.

## Methods and materials

### Study setting and interventions

The data was collected from trained peer educators of primary students (6–8 grade) in the Jimma zone of the districts of Shebe Senbo, Limmu Kossa, and Gera, where peer learning education interventions were implemented. Jimma zone is one of the administrative zones of the Oromia regional state located approximately 352 kilometers from Addis Ababa, the capital city of Ethiopia. The zone is generally located between 1,000 and 3,500 meters above sea level. The zone covers an area of 199,316 km2 with a total population of 2,770,329. The rural areas account for 89.1% of the population and urban areas account for 10.9 %. The zone is divided into 23 districts, with malaria-endemic areas in 17 % of the villages (kebeles) and 85 % of the zone's population living in malaria-risk areas. As part of large-scale school-based social and behavior change communication interventions on malaria prevention, an innovative and participatory approach called “PLEA-Malaria” was implemented in 75 rural primary schools in Jimma between 2017 and 2019.

### Study design, study period, and participants

A post-intervention cross-sectional study design was conducted using household surveys. The study was conducted from 2 April to 8 June 2020. All trained peer educators' students in grades 6–8 in Jimma Zone's 75 primary schools were source populations. Around 8,842 peer educators in 75 primary schools were trained in malaria prevention and control and have been carrying out malaria communication interventions at schools and in the community.

### Sample size

The sample size was determined by using a single population proportion formula:


$$\begin{array}{l} n=\left(Z\alpha /2\right)2\frac{P\left(1-P\right)}{d2}, \end{array}$$

where, *n* = desired sample size, *P* = 0.5 proportion of perceived performance of trained peer educators which indicates the maximum variability of the study population and gives maximum sample size, which was considered since there were no previous studies in Ethiopia that can especially help to address our objectives

z- Confidence interval – 95%,

d- Desired precision (%) – 5 % = 0.05,

Using the formula, the sample size was *n* = 384.

Since the source population is < 10,000, the population correction formula was used:


$$\begin{array}{l} n_{f}=\frac{n}{1+\frac{n}{N}},\;n_{f}=\frac{384}{1+\frac{384}{8842}}=\;368 \end{array}$$

where, ***n***_***f***_ = the final sample size, ***n*** = initial sample size (384), *N*; Source population of all trained peer Educator students = 8,842. In addition, 10% non-response was added. Finally, the calculated sample size became 404 trained peer educator students.

#### Sampling procedure

The study's target participants were trained peer educators from the Jimma zone's five districts, which included 75 primary schools. School focal teachers trained at least 8,842 primary peer educators (class representatives and 1–5 social network leaders) across 75 intervention schools. This totaled to 118 trained peer educators per school. Three districts were selected at random. In each district, ~15 primary schools were included in the intervention (*N* = 3^*^15^*^118 = 5,310), with 15 schools from all districts representing 30% and 5 schools from each district being included as well by using an equal allocation proportion. To obtain a sample size of 404 from 15 schools, ~27 peer educators from each school must be chosen. Finally, 27 peer educators were included, with 9 from each grade (6th, 7th, and 8th). Because the schools were closed due to COVID-19 during data collection, house-to-house data collection was conducted using a complete list of trained peer educators' students from each school as a sampling frame, taking COVID-19 preventive measures into account.

### Data collection and quality control

The questionnaire for addressing socio-demographic and psychographic factors was adapted from various studies (29, 30). In addition, questionnaires addressing intervention outcomes, perceptions, and peer education experiences were adapted from previous studies on program implementation documents (5, 29, 31, 32). Finally, the questionnaire containing the variables listed above on the malaria prevention and control program at school was used to collect data.

A pre-test on 5% of the sample was carried out. Following training, data were collected by health extension workers and teachers under the supervision of health professionals. Supervisors checked the data for completeness and consistency after each day of data collection.

### Operational definition and measurements

#### Perceived performance

The degree to which trained peer educator students believe that using a peer learning education approach would improve their performance in malaria prevention and control, as well as students' understanding of how well a peer learning education approach engaged in school-based malaria prevention and control functions (33, 34). The five points Likert scale was used and was computed. A high score indicates high perceived performance.

### Data processing and analysis

The data were validated, coded, and double-entered into the Epi Data 3.1 software package. The data was then exported to the SPSS 24.0 statistical package for analysis.

Statistical tests (Kolmogorov-Smirnov and Shapiro-Wilk tests) and visual evaluation of histograms and probability plots were used to assess the normality of the distribution of outcome variables. The Pearson correlation's major assumptions (normality and linearity of association) were tested.

Descriptive statistical measures such as mean, standard deviation, and frequency tables were done. Pearson's correlation analysis was carried out to examine the association between perceived performance and psychometric (acceptability, feasibility, and appropriateness) experiences again as well as with psychographic (knowledge, self-efficacy, risk perception, and attitude) predictors as bivariate analysis. Those variables which had significant associations with a perceived performance at p < 0.05 in the bi-variate analysis were qualified for multiple linear regression analysis. Finally, a multiple linear regression model was conducted to identify independent factors associated with the perceived performance of peer learning and education approach which was included in the final model. Regression coefficients (beta) with 95% CI were interpreted to understand the effects of predictors on the outcome variable.

## Result

### Socio-demographic characteristics

A total of 401 peer educators were involved in this study, making the response rate 99.2%. Of the total respondents, 242 (60.3 %) were male, and 229 (57.1 %) were between the ages of 15 and 19. The average age was 15.59 (standard deviation = 2.24). Most of them 340 (84.8 %) were rural residents, 295 (73.6 %) were Muslim, and roughly one-fifth of the respondents (72, 18.0 %) had taken health training other than malaria. Many respondents (344, 85.8 %) were from Oromo and the average family size of the respondents was 6.75 (SD 2.134) (Table 1).

**Table 1:** Socio demographic characteristics of the participants, Jimma zone, south west Ethiopia, 2020.

**Variables *n* = 401**	**Categories**	**Frequency**	**Percent (%)**
Place of residence	Urban	61	15.2
	Rural	340	84.8
Sex	Male	242	60.3
	Female	159	39.7
Age of students	10–14	144	35.9
	15–19	229	57.1
	20–24	28	7.0
Religion	Muslim	295	73.6
	Orthodox	72	18.0
	Protestant	34	8.5
Ethnic group of students	Oromo	344	85.8
	Amhara	31	7.7
	Kafa	17	4.2
	Other	9	2.2
Role in class	Class leader	69	17.2
	Vice leader	57	14.2
	Other	275	68.6
Health intervention training	Participate	72	18.0
other than malaria	Not participate	329	82.0
Latest average point	Excellent	48	12.0
	Very good	155	38.7
	Satisfactory	182	45.4
	Fair	14	3.5
	Poor	2	0.5

### Malaria related knowledge

The study found that almost all 395 respondents (98.5 %) had heard of malaria. The mean score of trained peer students for knowledge related to Essential Malaria Action was 0.6474 (SD = 0.1753). More than half of the students (212, 52.9) scored above the mean in EMA knowledge.

The majority of respondents (346, 86.0 %) reported fever as the main symptom of malaria, while six (1.5 %) did not know. Almost all (372, 92.8 %) respondents reported mosquito bites as the cause of malaria, while five (1.2%) did not know. About 377 respondents (94.0 %) reported that using a mosquito net can protect individuals from malaria, and less than five respondents did not know. Many respondents (342, 85.3 %) knew pregnant women and children under 3 years old were more at risk than others, while a few (17, 4.2 %) did not.

Drinking dirty water (82, 20.4 %), getting soaked in rain (78, 19.5 %), cold and changing weather (73, 18.2 %), shaking hands with someone who has malaria (40, 10.0 %), and eating sugarcane (29, 7.2 %) were cited as causes of malaria (Table 2).

**Table 2:** Frequency of the respondents' knowledge about malaria signs and symptoms, risks of transmission, and prevention methods in Jimma zone, south west, Ethiopia, 2020.

**Variables**		**Yes (f)**	**%**	**No**	**%**
Symptoms	Fever	346	86.3	55	13.5
	Feeling cold	224	55.9	177	44.1
	Headache	291	72.6	110	27.4
	Nausea and Vomiting	102	25.4	299	74.6
	Diarrhea	68	17.0	333	83.0
	Dizziness	106	26.4	295	73.6
	Loss of appetite	245	61.1	156	38.9
	Body ache or joint pain	175	43.6	226	56.4
	Pale eyes	93	23.2	308	76.8
	Body weakness	169	42.1	232	57.9
	I don't know	6	1.5	395	98.5
Causes malaria	Mosquito bites	372	92.8	29	7.2
	Eating sugarcane	29	7.2	372	92.8
	hunger (empty stomach)	97	24.2	304	75.8
	Drinking other dirty water	82	20.4	319	79.6
	Getting soaked with rain	78	19.5	323	80.5
	Cold or changing weather	73	18.2	328	81.8
	Lack of hygiene	144	35.9	257	64.1
	Shaking hands of person with malaria	40	10.0	359	89.5
	Don't know	5	1.2	396	98.8
Protect themselves against malaria	Sleep under a mosquito net	377	94.0	24	6.0
	Using repellants	71	17.7	330	82.3
	not staying out home at night	96	23.9	305	76.1
	Spray house with insecticide	236	58.9	165	41.1
	Keep house surroundings clean	192	47.9	209	52.1
	Fill in puddles (stagnant water)	229	57.1	172	42.9
	early diagnosis and treatment for fever	105	26.2	296	73.8
	Anti malarial drug compliance	246	61.3	155	38.7
	Don't know	2	0.5	399	99.5
Risk groups	Pregnant mother and child under 3 years	342	85.3		
	Adult women	17	4.2		
	Adult man	10	2.5		
	Child of six years old	15	3.7		
	Don't know	17	4.2		

### Parent and student communication

About 304 students (75.8 %) reported having discussed malaria with their families in the previous 12 months. Communication was reported to occur only on rare occasions by 203 (50.6 %) and 22 (5.5%), respectively. The topics of discussion included preventive methods, sleeping under ITN (174, 43.4 %), and fever treatment (104, 27.7 %) (Table 3).

**Table 3:** Parent student communication and family readiness in Jimma zone, Ethiopia 2020.

**Variables**	**Frequency**	**Percentage**
**Talk about malaria in general with parents in 12 months past**		
Yes No	304 97	75.8 24.2
**Frequency of talk about malaria with family**		
Only one day Rare Occasionally Always	22 47 203 32	5.5 11.7 50.6 8.0
**Point of discussion (communication)**		
ITN Fever treatment Anti malaria drug utilization Disposing stagnant water	174 104 111 150	43.4 25.9 27.7 37.4

### Peer education practice in school

Three hundred forty-one (85.0 %) students reported having conducted peer education in school in the previous 2 years, with the schedule being every 2 weeks, accounting for 124 students (30.9%). According to 156 (38.9 %) respondents, peer education was conducted in schools during the study period, while the majority (245, 61.1 %) reported that peer education was not conducted in their school during the study period (Table 4).

**Table 4:** Peer education conducted in two years past and currently conducted schedule in school in Jimma zone, Ethiopia 2020.

**Schedule**	**Past two years**		**Currently conducting**	
	**Frequency**	**Percent**	**Frequency**	**Percent**
Every day	14	3.5	14	3.5
Every week	90	22.4	30	7.5
Every two weeks	124	30.9	65	16.2
Every three weeks	9	2.2	4	1.0
Every four weeks	24	6.0	9	2.2
Only some times	80	20.0	34	8.5
Total conducted	341	85.0	156	38.9

### Descriptive statistics of perceived performance, psychographic, and team-level experience

The study found that the mean performance scale score was 44.31, which was higher than the expected mean value, and that more than half of the trained peer educator students (226, 56.4 %) scored higher than the mean value. Again, more than half of the trained peer educator students (231, 57.6 %) had a positive attitude toward malaria that was higher than the mean (38.54 ± 6.66), but nearly half of the students had a negative attitude toward malaria (202, 50.4%) and negative risk perception (205, 51.5%).

There were 15 items for the team-level experience. One weakly correlated item was excluded from the factors that emerged and 14 items were loaded under three factors, 6 for factor one, and 4 each for the remaining two factors. The mean score for experience of “eagerness to share” and “learn from each other” was 24.69 ± 3.76 and 16.23 ± 2.39, and 16.72 ± 2.53 for “comfortable in the team building process” and for “respect between team member experience” (Table 5).

**Table 5:** Descriptive statistics for dependent and independent variables of trained peer educator's student in Jimma zone.

**S.N**	**Variables**	**Number of items**	**Scale possible range**	**Observed range**	**Scale mean**	**SD**	**Cronbach's alpha**
1	Acceptability	7	7–35	11–35	26.76	4.35	0.74
2	Appropriateness	6	6–30	6–30	24.24	3.91	0.82
3	Feasibility	6	6–30	6–30	22.74	3.32	0.71
4	Perceived performance	11	11–55	11–55	44.31	6.53	0.84
5	Attitude	10	10–50	17–50	38.54	6.86	0.76
6	Self efficacy	10	10–50	18–50	41.14	4.89	0.85
7	Risk perception		5.5–27.5	7.5-25.5	18.75	3.06	
8	Eager to share and learn from each other	6	6–30	6–30	24.69	3.76	0.84
9	Team building process	4	4–20	8–20	16.23	2.39	0.72
10	Respectful between team members	4	4–20	7–20	16.72	2.53	0.74

### Association between dependent and continuous independent variables

The Pearson's correlation coefficients showed that other than attitude and the latest average, all continuous variables were significantly and positively correlated with perceived performance. The highest and lowest positive correlation was observed between perceived performance and appropriateness (*r* = 0.603, *p* < 0.01) and between perceived performance and knowledge for EMA (*r* = 0.172, *p* < 0.01) respectively. No strong multi-collinearity was observed between each independent variable. This correlation was required to decide whether to run the regression analysis or not (Table 6).

**Table 6:** Pearson's correlation between dependent with independent and socio demographic variables (*n* = 401).

**Components**	**1**	**2**	**3**	**4**	**5**	**6**	**7**	**8**	**9**	**10**	**11**	**12**	**13**	**14**
Perceived performance	1													
Age	0.195**	1												
Latest average point	0.030	0.001	1											
Family size	−0.194**	0.083	−0.031	1										
Acceptability	0.388**	0.202**	0.109*	−0.223**	1									
Appropriateness	0.603**	0.145**	0.034	−0.195**	0.556**	1								
Feasibility	0.600**	0.123*	0.053	−0.124*	0.475**	0.682**	1							
Self efficacy	0.489**	0.016	−0.030	−0.275**	0.303**	0.474**	0.422**	1						
Knowledge for EMA	0.172**	0.109*	−0.018	−0.022	0.239**	0.262**	0.173**	0.224**	1					
Attitude	0.068	−0.269**	−0.007	−0.084	0.091	0.049	0.140**	0.118*	0.108*	1				
Respective full between team member	0.522**	0.045	−0.054	−0.163**	0.248**	0.482**	0.473**	0.485**	0.244**	0.122*	1			
Eager to sharing Experience and learn from each other	0.586**	0.082	−0.036	−0.216**	0.300**	0.469**	0.420**	0.545**	0.269**	0.054	0.617**	1		
Happy to team formation process	0.520**	0.062	0.019	−0.118*	0.352**	0.545**	0.483**	0.442**	0.184**	0.055	0.633**	0.482**	1	
Risk perception	0.360**	0.404**	0.047	−0.086	0.193**	0.225**	0.155**	0.145**	0.070	−0.290**	0.151**	0.306**	0.241**	1

**Correlation is significant at the 0.01 level (2-tailed). *Correlation is significant at the 0.05 level (2-tailed).

### Independent predictors of performances of the school-based PLEA-malaria

In the bivariate analysis, all socio-demographic variables (age, sex, resident, family size, role in class, latest average point, favorite subject, previous training on other health issues, and grade level) were entered. From these variables, age, family size, and favorite subject were candidates for multiple linear regression analysis with a *p*-value < 0.05. From psychometric-related variables (acceptability, feasibility, and appropriateness), all were included for multiple linear regression. From psychographic-related variables (knowledge about EMA, attitude, risk perception, and self-efficacy), all, except attitude, were candidates for the multiple linear regression. From team-level experiences (experiences of being respectful to team members, eager to share and learn from each other, and happy in the team formation process), after conducting PCA, practicing peer education in school and communicating about malaria with family members were identified to be significantly associated with a *p*-value of < 0.05.

Prediction of perceived performances to PLEA in multiple linear regression indicated that with standardized regression coefficients, “eager to share experience and learn from each other” experience was found to be the best factor (β = 0.207, *p* < 0.05). This indicates that the “eager to share and learn from each other” experience increases the performance of trained peer educator students toward PLEA in school. This experience increases the performance of trained students toward malaria control and prevention by 20.7% keeping other conditions constant. Similarly, a unit-positive “think a risk perception about malaria” in trained students will increase the performances of individuals by 14.3 %, provided that all the other factors are kept unvaried. Similarly, in this study, self-efficacy toward conducting PLEA and favorite subjects (Afan Oromo and Chemistry) was found to increase performances of PLEA in malaria prevention and control by factors of β = 0.097 (*p* < 0.024) and β = 0.075 (*p* < 0.036), respectively (Table 7).

**Table 7:** Multivariable linear regression for perceived performance toward PLEA among Jimma zone trained peer educator students, south west Ethiopia, 2020.

**Variables**	**Standardized β**	***P*-value**	**95% CI for β**
Age	0.048	0.200	[−0.75, 0.355]
Family size	−0.025	0.490	[−0.290, 0.139]
Favorite of A/Oromo	−0.033	0.341	[−1.975, 0.684]
Favorite of chemistry	−0.058	0.102	[−2.712, 0.247]
Favorite of more than one subject*	0.075	0.036	[0.084, 2.511]
Knowledge of EMA	−0.050	0.160	[−0.309, 0.051]
Self efficacy*	0.097	0.024	[0.017, 0.242]
Feasibility*	0.253	0.000	[0.313, 0.682]
Acceptability	−0.003	0.953	[−0.129, 0.122]
Appropriateness*	0.163	0.002	[0.099, 0.442]
Peer education practice	0.021	0.550	[−0.861, 0.614]
Communicating with family	0.034	0.327	[−0.518, 1.553]
Risk perception*	0.143	0.000	[0.142, 0.466]
Respective full between team member experience	0.066	0.190	[−0.060, 0.301]
Happy to team formation process experiences	0.072	0.125	[−0.052, 0.424]
Eager to sharing experience and learn from each other*	0.207	0.000	[0.308, 0.826]

*Significant at p < 0.05.

## Discussion

The purpose of this study was to determine the perceived performance of PLEA and its associated, psychometric, psychographic, and socio-demographic factors of trained peer educators' students who used a peer learning education approach to improve their performance in malaria prevention and control activities.

This study found that the perceived performance score of PLEA-malaria among the key peer educators was 44.31(SD = 6.13). This is relatively higher than similar studies reported from England (35) and Eritrea (36). The difference may be due to the study setting as well as the study time that our study was completed later after due consideration had been given to peer learning and education approaches in most developed and developing countries. The perceived performance was the extent to which students believe that using a peer learning education approach improved their performance in malaria prevention and control. This implies that peer learning and education approach intervention in school was feasible and acceptable, indicating the program would likely produce the desired effect on malaria prevention and control practice. Some studies reported the feasibility of the peer education approach and indicated that feasibility of peer learning and education approach in school for interventional programs (37, 38), Another study from Moldova confirmed that the collaborative learning approach was well feasible in school (39). In this study, we found that the PLEA-malaria program was feasible through peer learning and education in schools which could successfully prevent and control malaria in students and their family.

In this study, having self-efficacy about the effect of school-based PLEA on malaria interventions was significantly and positively associated with perceived performances. This finding suggests that perceptions of one's confidence could improve their perceptions of the feasibility of PLEA-malaria implementation. Similar findings were reported from studies done on high schools in Western Cape, South Africa, where self-efficacy had a relationship with peer learning in schools (40). Similarly, a study conducted in Duzce, Turkey, found peer education implementation to increase the self-efficacy of individuals to undertake preventive action (41). Peer learning and education approach appears to contribute to the self-efficacy (confidence) of students to take preventive measures against malaria for themselves as well as their families and deliver malaria awareness messages both in school and in their community.

Risk perception regarding malaria was significantly and positively associated with the performance of PLEA on malaria prevention in schools. This finding suggests perceiving susceptibility and vulnerability to malarial infection has a positive impact on perceptions of performances of PLEA. Similar findings were reported from studies done in Zanzibar, Tanzania, where risk perception was positively associated with peer education program (42). Also, a study conducted in Rome, Italy, found a significant association between risk perception and the perception of peer educators (29). Similarly, a study conducted previously in Jimma also revealed the perceived risk of malaria influenced preventive practices of students that implemented the PLEA program and their community (28). Risk perceptions are central to malaria prevention and control behaviors of students such as ITN utilization, early medical consultation, disposal of stagnant water from their environment, and appropriate anti-malaria drug use.

This study showed that experiences relating to “eager to share and learn from each other” (team spirit) were significantly and positively associated with perceived performances from other team-level experiences. This finding is in line with studies undertaken in Eritrea on the effects of peer education on peer educators (36) and the finding from Australia that peer education program encourages individuals to share their experiences and support each other on matters relating to health (31). Good team spirit (sharing experiences) allows students to learn from each other about malaria preventive skills, working habits on peer learning activities in school, and emotional cues that result from a favorable attitude to the PLEA-malaria program.

The results of this study also revealed that of all the socio-demographic variables, the only variable that significantly predicted performances and perceptions of students regarding PLEA was the “favorite of more than one subject” (Afan Oromo and Chemistry). Trained peer educators who favored Afan Oromo and Chemistry positively predicted performances or belief that peer learning and education approach helped them, their peers, and their family with malaria prevention. This finding is similar to finding reported from related studies done at the University of Connecticut, US, exploring the relationship between the perception of peer learning and subject matters they prefer (43), and another study in Virginia, US, that revealed a relationship between favorite subjects and peer learning perception (44). Combining malaria prevention and control strategies and messages with Afan Oromo and Chemistry subjects while implementing the schools-based peer learning and education approach using students as change agents would enhance the effectiveness of the PLEA-malaria program. This can lead to meeting the targets of the national malaria elimination program.

## Conclusion

The result of this study suggests that the peer educators perceived the school-based PLEA-malaria was appropriate and effectively implemented. Thus, it is recommended to consider personal and social (team) factors such as self-efficacy, risk perceptions, and peer education team spirits when designing and implementing such programs in schools. Further research involving complex modeling could be conducted to understand the mechanism by which the PLEA implementation influences malaria preventive behaviors among all students.

### Limitations of the study

This study had strength by considering malaria prevention and control strategy from the perspective of individual behavioral performance indicators required during the period of malaria elimination-eradication plans. Schools and school-based approaches are currently getting global attention for involvement in public health. However, the study has a few limitations. Given the nature of the cross-sectional study design, it was difficult to establish the cause-effect relationship between the associated factors and outcome variables. Recall bias may be one of the limitations of the study and interviewer bias may also have occurred. Data was collected based on participants' self-reports, which may be associated with socially desirable bias.

## Data availability statement

The original contributions presented in the study are included in the article/supplementary material, further inquiries can be directed to the corresponding author/s.

## Ethics statement

The studies involving human participants were reviewed and approved by Institute of Public Health, Jimma University Institutional Review Committee. The patients/participants provided their written informed consent to participate in this study.

## Author contributions

ADer and FA conceived the idea, conceptualized it, undertook the investigation and data analysis, and wrote the manuscript. ADer, ADeb, AE, and MG reviewed and edited the final draft, contributed to the methodology, and drafted the manuscript. All authors gave their final approval of the version of the manuscript submitted for publication.

## Funding

This work was supported financially by Jimma University. However, the University had no role in the study design, data collection, analysis, decision to publish, or preparation of the manuscript.

## Conflict of interest

The authors declare that the research was conducted in the absence of any commercial or financial relationships that could be construed as a potential conflict of interest.

## Publisher's note

All claims expressed in this article are solely those of the authors and do not necessarily represent those of their affiliated organizations, or those of the publisher, the editors and the reviewers. Any product that may be evaluated in this article, or claim that may be made by its manufacturer, is not guaranteed or endorsed by the publisher.

## Abbreviations

ACP: Advancing Community's Practice
BCC: Behavioral change communication
EMA: Essential Malaria Activities
IEC: Information education communication
PEs: Peer educators
PLEA: peer learning and education approach
SBCC: Social and behavioral change communication.

## References

[1] Christianson A Howson CP Modell B. March of dimes. Global report on birth defect. The Hidden Toll of Dying and Disabled Children. New York. (2006). p. 10–6.17728910

[2] World Health Organization. Global Malaria Programme. A framework for malaria elimination. Geneva World Heal Organ. (2017) 100. Available online at: http://apps.who.int/iris/bitstream/handle/10665/254761/9789241511988-eng.pdf?sequence=1 (accessed January, 2020).

[3] World Health Organization (WHO). World Malaria Report 2018. Geneva: World Health Organization (WHO) (2018).

[4] World Health Organization (WHO). World Malaria Report 2021. Geneva: World Health Organization (WHO) (2021).

[5] USAID US precidantial initiative Presedent M. Ethiopia Malaria Operational Plan FY 2019- President's Malaria. (2019) 1–71. Available online at: https://reliefweb.int/report/ethiopia/president-s-malaria-initiative-ethiopia-malaria-operational-plan-fy-2019

[6] Autino B Noris A Russo R Castelli F. Epidemiology of malaria in endemic areas. Mediterr J Hematol Infect Dis. (2012) 4:60. 10.4084/mjhid.2012.060 23170189 PMC3499992

[7] Gari T Loha E Deressa W Solomon T Lindtjørn B. Malaria increased the risk of stunting and wasting among young children in Ethiopia: results of a cohort study. PLoS ONE. (2018) 13:983. 10.1371/journal.pone.0190983 29324840 PMC5764317

[8] Clarke SE Rouhani S Diarra S Saye R Bamadio M Jones R. Impact of a malaria intervention package in schools on Plasmodium infection, anaemia and cognitive function in schoolchildren in Mali: a pragmatic cluster-randomised trial. BMJ Global Health. (2017) 2:e000182. 29081992 10.1136/bmjgh-2016-000182 PMC5656118

[9] Clarke SE Rouhani S Diarra S Saye R Bamadio M Jones R. Impact of a malaria intervention package in schools on Plasmodium infection, anemia and cognitive function in schoolchildren in Mali: a pragmatic cluster-randomized trial. BMJ Glob Heal. (2017) 2:182. 10.1136/bmjgh-2016-000182 29081992 PMC5656118

[10] Kuecken M Thuilliez J Valfort M-A. Does malaria control impact education? A study of the global fund in Africa. Doc Trav du Cent d'Economie la Sorbonne. (2013) 75:23–33.

[11] Pullan RL Bukirwa H Staedke SG Snow RW Brooker S. Plasmodium infection and its risk factors in eastern Uganda. Malar J. (2010) 9:2. 10.1186/1475-2875-9-2 20044942 PMC2822788

[12] Ashton RA Kefyalew T Tesfaye G Pullan RL Yadeta D Reithinger R. School-based surveys of malaria in Oromia Regional State, Ethiopia: a rapid survey method for malaria in low transmission settings. Malar J. (2011) 10:1025. 10.1186/1475-2875-10-25 21288368 PMC3039636

[13] World Health Organization (WHO). Ethiopian health sector transformation plan.2015/16 - 2019/20. Fed Democr Repub Ethiop Minist Heal. (2015) 20:50.

[14] Deribew A Dejene T Kebede B Tessema GA Melaku YA Misganaw A. Incidence, prevalence and mortality rates of malaria in Ethiopia from 1990 to 2015: analysis of the global burden of diseases 2015. Malar J. (2017) 16:1914. 10.1186/s12936-017-1919-4 28676108 PMC5496144

[15] Umwangange ML Chironda G Mukeshimana M. Knowledge, attitude, and practice towards malaria prevention among school children aged 5−14 years in Sub-Saharan Africa - a review of literature knowledge, attitude and practice towards malaria prevention among school children aged 5−14 years in Sub-S. (2018) 5:4. 10.4314/rjmhs.v1i1.4

[16] Debela Y. Malaria related knowledge and child to parent communication regarding prevention and control of malaria among primary school students in Jimma Zone, South West Ethiopia. Am J Heal Res. (2014) 2:284. 10.11648/j.ajhr.20140205.20

[17] Mbaabu Lairumbi Patrick Mbindyo and Timothy Abuya. End of Project Evaluation of the School Health Malaria Control Initiative (SMHCI). (2017). Available online at: http://hdl.handle.net/123456789/2729

[18] World Health Organization. Global school health initiatives: achieving health and education outcomes. In Report of a Meeting. Bangkok (2015). p. 23–5.

[19] Rosado JL González KE Caamaño MC García OP Preciado R Odio M. Efficacy of different strategies to treat anemia in children : a randomized clinical trial. Nutri J. (2010) 9:1–10. 10.1186/1475-2891-9-40 20863398 PMC2955680

[20] President's Malaria Initiative Ghana Health Services Ghana Education services John Hopkins Center for Communication programs (2016). Promoting Malaria Prevention Through Primary Schools: Communication Guide for Teachers. (2016).

[21] Moonen B Cohen JM Snow RW Slutsker L Drakeley C Smith DL. Operational strategies to achieve and maintain malaria elimination. The Lancet. (2010) 376:1592–603. 21035841 10.1016/S0140-6736(10)61269-X PMC3037542

[22] Khasakhala L Otido J Crudder C Foundation MH Diseases I. Group E. Spons Doc From. (2008) 372:127–38. 10.1016/S0140-6736(08)61034-X 18620950 PMC2495044

[23] Organization WH. Global School Health Initiatives: Achieving Health and Education Outcomes: Report of a Meeting, Bangkok, Thailand, 23–25 November 2015. Bangkok: World Health Organization (2017).

[24] Gray G Barnekow Rasmussen V Young I for Europe WHORO of of Health Promoting Schools. International Planning Committee EN. Health-Promoting Schools : A Practical Resource for Developing Effective Partnerships in School Health, Based on the Experience of the European Network of Health Promoting schools / by Gay Gray, Ian Young and Vivian Barnekow. Copenhagen : WHO Regional Office for Europe (2006). p. EUR/06/5061578.

[25] Brieger WR Delano GE Lane CG Oladepo O Oyediran KA. West African youth initiative: outcome of a reproductive health education program. J Adolesc Heal. (2001) 29:436–46. 10.1016/S1054-139X(01)00264-6 11728893

[26] Ajuwon AJ Brieger WR. Evaluation of a school-based Reproductive Health Education Program in rural South Western, Nigeria. Afr J Reprod Health. (2007) 11:47–59. 10.2307/25549715 20690287

[27] Lock K Walker L Cain M Walters L. The experiences of youth peer educators sharing health information. In 12th National Rural Health Conference. Adelaide, Australia (2013) 1–9.35363974

[28] Ayi I Nonaka D Adjovu JK Hanafusa S Jimba M Bosompem KM. School-based participatory health education for malaria control in Ghana: engaging children as health messengers. Malar J. (2010) 9:98. 10.1186/1475-2875-9-98 20398416 PMC2865503

[29] Kebede Y Abebe L Alemayehu G Sudhakar M Birhanu Z. School-based social and behavior change communication (SBCC) advances community exposure to malaria messages, acceptance, and preventive practices in Ethiopia : a pre- posttest study. (2020) 54:5189. 10.1371/journal.pone.0235189 32584891 PMC7316301

[30] Abdi F Simbar M. The peer education approach in adolescents- Narrative review article. Iran J Public Health. (2013) 42:1200–6. 26171331 PMC4499060

[31] Kim CR Free C. Recent evaluations of the peer-led approach in adolescent sexual health education: a systematic review. Int Fam Plan Perspect. (2008) 34:89–96. 10.1363/3408908 18644760

[32] Lock K Walker L Cain M Walters L. The experiences of youth peer educators sharing health information. In 12th National Rural Health Conference. Adelaide, Australia. (2015) 1–9.35363974

[33] Singh K Kaur S. Psychological empowerment of teachers: Development and validation of multi-dimensional scale. Int J Recent Technol Eng. (2019) 7:340–7.

[34] de Menezes S Premnath D. Near-peer education: a novel teaching program. Int J Med Educ. (2016) 7:160–7. 10.5116/ijme.5738.3c28 27239951 PMC4885635

[35] Weiner BJ Lewis CC Stanick C Powell BJ Dorsey CN Clary AS. Psychometric assessment of three newly developed implementation outcome measures. Implement Sci. (2017) 12:6353. 10.1186/s13012-017-0635-3 28851459 PMC5576104

[36] Eisenstein C Zamperoni V Humphrey N Deighton J Wolpert M Rosan C. Evaluating the peer education project in secondary schools. J Public Ment Health. (2019) 18:58–65. 10.1108/JPMH-07-2018-0048

[37] Andrews M Manning N. A Study of Peer Learning in the Public Sector. (2015) (September).

[38] Hu P Han L Sharma M Zeng H Zhang Y Li. H. Evaluation of Cognitive and Behavioral Effects of Peer Educa- tion Model-Based Intervention to Sun Safe in Children. Iranian J Public Health. (2014) 43:300–9. PMC4419167 25988089

[39] Karki P Prabandari YS Probandari A Banjara MR. Feasibility of school-based health education intervention to improve the compliance to mass drug administration for lymphatic Filariasis in Lalitpur district, Nepal: a mixed methods among students, teachers and health program manager. PLoS ONE. (2018) 13:e0203547. 10.1371/journal.pone.0203547 30216390 PMC6138383

[40] Lesco G Squires F Babii V Bordian N Cernetchi O Martin Hilber A. The feasibility and acceptability of collaborative learning in improving health worker performance on adolescent health: findings from implementation research in Moldova. BMC Health Serv Res. (2019) 19:2. 10.1186/s12913-019-4158-2 31138177 PMC6537176

[41] Timol F Vawda MY Bhana A Moolman B Makoae M Swartz S. Addressing adolescents' risk and protective factors related to risky behaviors: findings from a school-based peer-education evaluation in the Western Cape. J Soc Asp of HIV/AIDS. (2016) 13:197–207. 10.1080/17290376.2016.1241188 27892820 PMC5349190

[42] Karaca A Akkus D Sener DK. Peer education from the perspective of peer educators. J Child Adolesc Subst Abus. (2018) 27:76–85. 10.1080/1067828X.2017.1411303

[43] Bauch JA Gu JJ Msellem M Mårtensson A Ali AS Gosling R. Perception of malaria risk in a setting of reduced malaria transmission: a qualitative study in Zanzibar. (2013) 12:1–10. 10.1186/1475-2875-12-75 23433302 PMC3584900

[44] Mikami AY Ruzek EA Hafen CA Gregory A Joseph P. Brunswick N. HHS public access. Ann Glob Health Author Manuscript. (2017) 46:2341–54. 10.1007/s10964-017-0724-2 28755252 PMC5671357

